# Mobile Phone-Based Lifestyle Intervention for Reducing Overall Cardiovascular Disease Risk in Guangzhou, China: A Pilot Study

**DOI:** 10.3390/ijerph121215037

**Published:** 2015-12-17

**Authors:** Zhiting Liu, Songting Chen, Guanrong Zhang, Aihua Lin

**Affiliations:** 1 Department of Medical Statistics and Epidemiology, School of Public Health, Sun Yat-sen University, Guangzhou 510080, China; liuzhiting6@163.com; 2 Department of Infection Management, First Affiliated Hospital of Kunming Medical University, Kunming 650032, Yunnan, China; chengsongting2015@163.com; 3 Health Management Center, Guangdong General Hospital, Guangdong Academy of Medical Sciences, Guangzhou 510180, China; gavincheung@yeah.net

**Keywords:** cardiovascular disease risk, mobile phone-based intervention, middle-aged and older adults, China

## Abstract

With the rapid and widespread adoption of mobile devices, mobile phones offer an opportunity to deliver cardiovascular disease (CVD) interventions. This study evaluated the efficacy of a mobile phone-based lifestyle intervention aimed at reducing the overall CVD risk at a health management center in Guangzhou, China. We recruited 589 workers from eight work units. Based on a group-randomized design, work units were randomly assigned either to receive the mobile phone-based lifestyle interventions or usual care. The reduction in 10-year CVD risk at 1-year follow-up for the intervention group was not statistically significant (–1.05%, *p* = 0.096). However, the mean risk increased significantly by 1.77% (*p* = 0.047) for the control group. The difference of the changes between treatment arms in CVD risk was –2.83% (*p* = 0.001). In addition, there were statistically significant changes for the intervention group relative to the controls, from baseline to year 1, in systolic blood pressure (–5.55 *vs.* 6.89 mmHg; *p* < 0.001), diastolic blood pressure (–6.61 *vs.* 5.62 mmHg; *p* < 0.001), total cholesterol (–0.36 *vs.* –0.10 mmol/L; *p* = 0.005), fasting plasma glucose (–0.31 *vs.* 0.02 mmol/L; *p* < 0.001), BMI (–0.57 *vs.* 0.29 kg/m^2^; *p* < 0.001), and waist hip ratio (–0.02 *vs.* 0.01; *p* < 0.001). Mobile phone-based intervention may therefore be a potential solution for reducing CVD risk in China.

## 1. Introduction

Cardiovascular disease (CVD) remains the leading cause of death in adults on a world scale [1], and is responsible for about 40% of deaths in China [2]. Because of the increasing disease burden of CVD in the past several years, interventions targeting CVD risk reduction are urgent public priorities. There is emerging strong evidence that not only drug treatment targeting risk factors but also unhealthy lifestyle change is an effective way in CVD primary prevention [3,4,5,6,7]. However, cardiovascular risk reduction programs often need much work from the providers [8] and they have not developed to reach a wide at-risk population [9].

In response to these gaps, several researchers have proposed that mobile phone-based intervention for chronic diseases would be a promising way to address access, coverage, and equity gaps in developing countries and low-resources settings [10]. The phones can prompt for action or information, display multi-media content, send and receive data to and from the internet—all features with potential for delivering a cardiovascular risk reduction program. Mobile phones are playing an important role in healthcare service because they can identify patients at risk for developing health problems, provide tailored and frequent health education and assist patients in adopting a healthy lifestyle [11,12]. Previous studies have indicated that even in some developing regions, most patients have access to mobile technology, and most are willing to participate in automatic telephone disease management support [13]. Mobile phone-based programs targeting CVD risk reduction would be a cost-effective method in countries with lower resources, such as China. However, reports of mobile phone-based intervention for reducing CVD risk in China remain limited.

Several studies in the United States and Europe have indicated that the CVD risk assessment and communication could increase patients’ intent to initiate risk factors management and enhance the effectiveness of treatment [14,15,16,17]. CVD is the result of the interaction of multiple risk factors [18,19]. Large epidemiological studies have shown that cardiovascular disease risk could be better understood and perceived by calculating the future risk of cardiovascular events through weighting each individual risk factor a relative contribution [20]. In China, the cardiovascular risk prediction model has been developed, which is called “the assessment method of onset risk of ischemic cardiovascular disease (ICVD) within 10 years” [21]. This prediction equation provides an opportunity to better estimate the overall CVD risk.

Therefore, we developed a mobile phone-based intervention program to reduce CVD risk, which was assessed by the Chinese cardiovascular disease risk assessment method. The intervention included the CVD risk assessment and communication, and delivering tailored health education to subjects by mobile phone. We hypothesized that relative to the control group, mobile phone-based intervention would reduce the overall CVD risk.

## 2. Method

### 2.1. Study Overview

We conducted this study at the health management center of a hospital in Guangzhou, China. The aim of this study was to determine the effectiveness of risk evaluation and mobile phone-based intervention program for modifying risk factors and unhealthy lifestyle so as to promote reduction of overall CVD risk. This study was a cluster randomized controlled program. From work units, whose staff were allocated to have medical examinations between October and December 2012 at the health management center of Guangzhou General Hospital of Guangzhou Command, eight were selected for inclusion in the study; five work units were randomly assigned to the intervention group and three were assigned to the usual medical examination group. A work unit is the name given to a place of employment in the People’s Republic of China, and employees could have the annual medical examination arranged by their work units. Participants were enrolled between October and December 2012 and were allocated to the mobile phone-based intervention group or the usual medical examination group. Study outcomes were assessed at 12-month follow-up.

### 2.2. Ethics Statement

This study was approved by the Institutional Review Board of the School of Public Health, Sun Yat-sen University. Written informed consent was obtained from all the study participants. (Trial registration: ChiCTR-TRC-13003831).

### 2.3. Participants and Enrollment

Work units whose employees had been allocated to have a medical examination at the Guangzhou General Hospital of Guangzhou Command for more than 2 years at the baseline were eligible for the study. Participants without known cardiovascular disease from the selected work units were eligible for enrolment if they were aged 45–75 years and willing to participate in the program. Exclusion criteria were a history of mental abnormalities, having difficulty in communication, such as reading or answering the questionnaire, unable to understand the aim of this study, currently participating in another clinical trial or had done so within the previous 6 months.

### 2.4. Randomization and Masking

A list of the names of work units, whose employees were allocated to have a medical examination in the Guangzhou General Hospital during October to December 2012, was obtained by the research team. From the 18 eligible work units at baseline, eight work units, whose random numbers were smallest, were randomly selected into this study. Then they were randomized into the intervention group or the usual medical examination group according to the random number. If the random number was odd, the work unit would be allocated to the control group; otherwise, the work unit would be allocated to the intervention group. The randomization was done via a computerized procedure. Neither participants nor investigators were masked to group assignment in this study.

### 2.5. Intervention

The intervention group received a computerized CVD risk evaluation, follow-up phone calls and text messages targeting reducing the CVD risk in addition to the usual medical examination. This program was developed by a team of health education expert, cardiologist and field health worker.

In order to facilitate the risk evaluation and individualized intervention for each participant, we developed an individualized electronic health prescription software (IEHPS). According to one’s demographic characteristics and physical examination results, the IEHPS could achieve the following functions: (1) Calculate participants’ overall risk of CVD in the next ten years, and provide the average risk and optimal risk at the same age. The individual’s risk, average risk and optimal risk were displayed by a bar chart at the same time. The average risk refers to the average risk of the same age, and the optimal risk denotes the risk of those who are non-smoker, non-diabetes of the same age and sex, with systolic blood pressure lower than 120 mmHg, total cholesterol lower than 5.17 mmol/L and body mass index lower than 24 kg/m^2^; (2) Inform of one’s present abnormal physical examination index, CVD risk factors, and unhealthy lifestyles; (3) Provide one’s individualized intervention plan. The plan included guidance of healthy lifestyle, improvement targets for risk factors and drug treatment goals for those being treated.

Participants randomized to the intervention group received an individualized electronic prescription, printed out by the IEHPS, and a handbook for the cardiovascular health education within two weeks after the baseline medical examination. The health education contents of the handbook were derived from the Chinese guidelines for prevention of cardiovascular diseases [22] and the PREMIER Trial [23], in which an intensive lifestyle intervention successfully reduced the coronary heart disease risk. They included the demonstrations of a healthy dietary patterns and cooking methods (e.g., decreasing the use of salt and cooking oil, and increasing vegetable and fruit consumptions), increasing physical activity, maintaining a healthy weight, quitting smoking, reducing excessive alcohol intake and keeping a healthy psychological condition. In addition, participants in the intervention group received a 15-min face to face counseling with a trained field health worker when they enrolled to the study. The counseling program consisted of four aspects: (1) Inform participants of their estimated 10-year risk of CVD, make interpretations about what is the overall risk of CVD events in the next 10 years and how the risk score is calculated; (2) Educate participants about the personalized modifiable risk factors, and the potential benefits of risk-reducing strategies; (3) Explain the individualized electronic prescription generated by the EHPS; (4) Understand the barriers participants may encounter in the process of implementing each chosen risk-reducing strategy, and encourage them to overcome those difficulties.

During the next 12 months, participants in the intervention group received follow-up phone calls and text messages sent by the research team. The frequency of phone calls and text messages was based on the participants’ CVD risk level (Table 1). Phone calls lasted about 5 to 8 min, and were used to deliver the counseling program. The counseling program: (1) inquired the situation of the intervention implementation; (2) informed the participants about the personalized modifiable risk factors and the possible benefits of risk-reducing strategies; and (3) provided the guidance of a healthy lifestyle, which focused on the changes in diet, physical activity, smoking cessation, limits on alcohol consumption and stress management. Messages were aimed to improve the maintenance of the participants by reminding their risk factors and encouraging them to renew their commitment to CVD prevention.

**Table 1:** The frequency of phone calls and text messages.

10-Year Risk of CVD	Risk Classification	Frequency of Phone Calls	Frequency of Text Message Sending
<5%	Very low risk	Twice per month	once per month
5%≤ & <10%	Low risk	Twice per month	once per month
10%≤ & <20%	Moderate risk	Twice per month	Twice per month
20% ≤& <40%	High risk	Three times per month	Three times per month
≥40%	Very high risk	Once per week	Once per week

### 2.6. Control Group

Participants in the control group received the annual medical examination with a usual medical report. This report included the results of physical examination and the normal values of the indicators. Apart from these contents, no other lifestyle promotion services were provided.

### 2.7. Outcomes and Measures

The primary outcome of the study was the change in the estimated CVD risk between baseline and follow-up point (12 months). The CVD risk was evaluated by “the assessment method of onset risk of ischemic cardiovascular disease within 10 years”, which was initiated by the national science and technology research team in China [21]. This tool is developed on the basis of cardiovascular disease risk prediction model in Europe and America, and also considers the risk factor patterns and the profile of CVD in China. The prediction model estimates CVD risk from age, systolic blood pressure, body mass index (BMI), total cholesterol (TC), diabetes and smoking, with ischemic cardiovascular disease (ICVD; including coronary heart disease and ischemic stroke) as the end event (Supplementary Table S1). The model has been proved to be a well-validated tool for the prediction of the cardiovascular risk in Chinese population [21]. In addition, we analyzed the changes in the components of the risk score.

Secondary outcomes included the diastolic blood pressure (DBP), triglyceride (TG), high density lipoprotein (HDL) cholesterol, low density lipoprotein (LDL) cholesterol, fasting plasma glucose (FPG) and waist hip ratio (WHR).

Blood pressure was measured with an automatic Omron sphygmomanometer in a sitting position after 5 min of rest, and the mean of two measurements taken 2 min apart was used. Hypertensive was defined as systolic BP ≥ 140 mmHg and/or diastolic BP ≥ 90 mmHg, and/or currently taking anti-hypertensive medications. Weight and height were measured on a digital scale to the nearest 0.5 kg or centimeter in light clothes and without shoes. Waist circumference and hip circumference were measured by trained investigators to the nearest 0.1 cm. We measured smoking status by self-report. Current smoking was defined as at least one cigarette a day for 6 months or more. Blood samples were collected after a 10 h overnight fast. Glucose, total cholesterol, HDL cholesterol, LDL cholesterol, and triglyceride were measured without sample pre-treatment. Fasting glucose was measured by the hexokinase enzymatic method. Participants with a FPG level ≥ 7.0 mmol/L and/or self-reported current use of anti-diabetic medications were diagnosed as diabetics. Total cholesterol and triglyceride were determined by automated enzymatic methods. LDL-C and HDL-C levels were tested by homogeneous methods. All outcomes were measured at the baseline and 12 months in both study arms. The baseline and follow-up assessments were conducted by the well-trained medical students and all the laboratory tests were carried out in the health management center of the Guangzhou General Hospital of Guangzhou Command.

### 2.8. Sample Size

Based on the assumption of an SD of 8 units and 20% dropout, a sample size of 406 (203 per group) was required to detect a difference of 2.5% in the changes of risk value between groups with 80% power. The type-I error rate was 0.05.

### 2.9. Statistical Analysis

An intention-to-treat approach was used for all participants in the intervention and control group, and all missing data were imputed. The multiple imputation by Markov chain Monte Carlo (MCMC) method was applied and we generated 15 imputations to replace missing values for variables. We summarized baseline sample characteristics using descriptive statistics and compared groups using *t* or χ^2^ tests. The primary outcome and secondary outcomes were examined using paired *t* tests for changes within each intervention arm. Additional analyses were conducted to compare the mean changes in outcomes between arms using the linear mixed model, adjusting for work units, the baseline value of the outcome and variables deemed relevant to behavior change a priori or that differed between groups at baseline (*p* < 0.05). Subgroups analyses were based on the sex, age (60 years as threshold) and baseline 10-year risk of CVD (5% as threshold). We also conducted sensitivity analyses on the completion population, including all participants for whom data were available at all study assessments. All statistical analyses were carried out in SAS version 9.2. Two-side *p* values were reported with a statistical significance level of <0.05.

## 3. Results

### 3.1. Baseline Characteristics and Follow-Up

**Figure 1:**
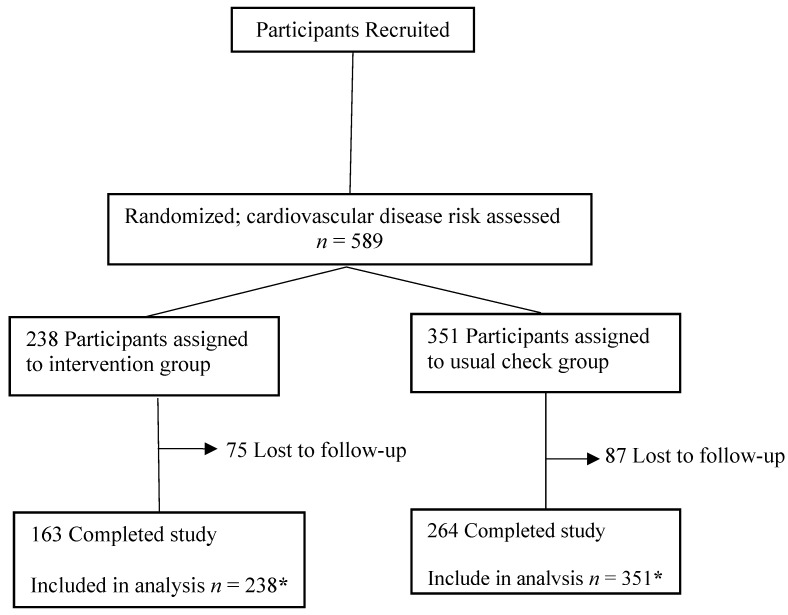
Study design and participant flow. * Data were imputed for the participants lost to follow-up.

As is shown in Figure 1, 589 participants from eight work units were recruited to the study and were assigned to the intervention group (*n* = 238) or the control group (*n* = 351), of whom 427 (72.5%) attended the follow-up visit at 12 months.

Table 2 lists baseline characteristics of the 589 study participants. The average age was 60.57 years, 41.8% were female, 52.6% had a college education level or above and 43.1% had income greater than 5000 RMB per month. Mean (SD) systolic and diastolic BP were 128.16 (13.40) and 78.25 (11.00) mmHg, and the mean (SD) FPG was 5.55 (1.31) mmol/L. The baseline prevalence rates of hypertensive and diabetic were 24.6% and 7.8%, respectively. There were no statistically significant differences between intervention participants and controls in most baseline characteristics and measurements, except that participants in the intervention group were younger (58.72 *vs.* 61.82, *p* < 0.001) and more likely to have lower monthly income level (*p* = 0.001).

Four hundred and twenty seven participants (72.5%) completed the 12-month follow-up, with no significant difference between group allocations. The participants who did not return for follow-up at 12 months were similar to the retained cases in terms of most baseline characteristics but were more likely to be younger, male, current smoker and have a higher level of TC (Supplementary Table S2).

### 3.2. Primary Study Outcome: 10-Year Risk of CVD

The mean (SD) baseline 10-year risk of CVD was 6.66 (7.66) %. Changes in 10-year risk of CVD from baseline to follow-up, by the treatment arms, are listed in Table 3. The mean risk increased significantly between baseline and 12 months by 1.77% (95%CI: 0.62% to 2.92%) for participants in the control group. For those in the intervention group, the reduction of 10-year risk of CVD was not statistically significant (–1.05%, *p* = 0.096). The adjusted difference of the changes between treatment arms was –2.83% (95%CI: −4.47% to −1.18%, *p* = 0.001).

**Table 2:** Baseline participant characteristics (*n* (%)/Mean ± SD).

Characteristic	Total Sample	Intervention Group	Control Group	*p*
Age	60.57 ± 8.97	58.72 ± 8.92	61.82 ± 8.80	<0.001
Female	246 (41.8)	99 (41.6)	147 (41.9)	0.945
Married	569 (96.6)	233 (97.9)	336 (95.3)	0.153
Education				
Middle School Or Lower	136 (23.1)	47 (19.8)	89 (25.4)	0.268
Senior High School	143 (24.3)	62 (26.1)	81 (23.1)	
College Or Above	310 (52.6)	129 (54.2)	181 (51.6)	
Personal Monthly Income				
<¥3000	176 (29.9)	83 (34.9)	93 (26.5)	0.001
¥3000~	159 (27.0)	75 (31.5)	84 (23.9)	
¥5000~	254 (43.1)	80 (33.6)	174 (49.6)	
Current Smoker	130 (22.1)	57 (24.0)	73 (20.8)	0.365
Alcohol Use	154 (26.2)	69 (29.0)	85 (24.2)	0.196
BMI, kg/m^2^	24.05 ± 3.12	23.77 ± 3.21	24.24 ± 3.05	0.076
WHR	0.89 ± 0.05	0.89 ± 0.06	0.89 ± 0.05	0.540
SBP, mmHg	128.16 ± 13.40	128.60 ± 14.10	127.90 ± 12.92	0.536
DBP, mmHg	78.25 ± 11.00	78.54 ± 10.25	78.06 ± 11.49	0.602
FPG, mmol/L	5.55 ± 1.31	5.57 ± 1.50	5.54 ± 1.16	0.769
TC, mmol/L	5.63 ± 1.03	5.63 ± 1.02	5.63 ± 1.04	0.954
triglyceride, mmol/L	1.80 ± 1.20	1.83 ± 1.37	1.77 ± 1.07	0.565
LDL, mmol/L	3.61 ± 0.89	3.61 ± 0.84	3.61 ± 0.92	0.999
HDL, mmol/L	1.71 ± 0.37	1.68 ± 0.35	1.72 ± 0.38	0.154
Hypertensive	145 (24.6)	49 (20.6)	96 (27.4)	0.062
Diabetic	46 (7.8)	13 (5.5)	33 (9.4)	0.080

**Table 3:** Changes in outcomes (Mean (95%CI)).

Outcome	Intervention Group			Control Group			Crude Effect Size ^a^	Adjusted Effect Size ^b^
	Baseline	Year 1	Change	Baseline	Year 1	Change		
10-year risk of CVD, %	5.82	4.76	−1.05	7.22	9.00	1.77	−2.83	−2.83
	(4.93 to 6.69)	(3.41 to 6.11)	(−2.32 to 0.22)	(6.39 to 8.08)	(7.81 to 10.19)	(0.62 to 2.92)	(−4.52 to −1.13)	(−4.47 to −1.18)
Components of Risk Score								
SBP, mmHg	128.58	123.02	−5.55	127.88	134.77	6.89	−12.45	−12.45
	(126.78 to 130.37)	(120.67 to 125.37)	(−7.70 to −3.41)	(126.53 to 129.23)	(132.97 to 136.57)	(5.17 to 8.61)	(−15.09 to −9.80)	(−15.02 to −9.87)
TC, mmol/L	5.63	5.27	−0.36	5.63	5.52	−0.10	−0.26	−0.26
	(5.50 to 5.76)	(5.12 to 5.42)	(−0.51 to −0.21)	(5.52 to 5.74)	(5.37 to 5.67)	(−0.25 to 0.04)	(−0.45 to −0.07)	(−0.44 to −0.08)
BMI, kg/m^2^	23.77	23.20	−0.57	24.24	24.52	0.29	−0.86	−0.86
	(23.37 to 24.18)	(22.73 to 23.68)	(−1.00 to −0.14)	(23.92 to 24.56)	(24.10 to 24.94)	(−0.08 to 0.66)	(−1.34 to −0.38)	(−1.32 to −0.39)
Other Outcomes								
DBP, mmHg	78.54	71.94	−6.61	78.06	83.68	5.62	−12.23	−12.23
	(77.24 to 79.84)	(70.34 to 73.53)	(−8.14 to −5.07)	(76.86 to 79.26)	(82.41 to 84.95)	(4.39 to 6.84)	(−14.12 to −10.33)	(−14.03 to −10.43)
FPG, mmol/L	5.57	5.28	−0.31	5.54	5.55	0.02	−0.32	−0.32
	(5.38 to 5.76)	(5.10 to 5.45)	(−0.49 to −0.12)	(5.41 to 5.66)	(5.42 to 5.69)	(−0.13 to 0.16)	(−0.52 to −0.12)	(−0.51 to −0.13)
TG, mmol/L	1.83	1.74	−0.10	1.77	1.64	−0.13	0.04	0.04
	(1.66 to 2.01)	(1.52 to 1.95)	(−0.31 to 0.12)	(1.60 to 1.89)	(1.51 to 1.78)	(−0.28 to 0.01)	(−0.20 to 0.27)	(−0.19 to 0.26)
HDL, mmol/L	1.68	1.52	−0.16	1.72	1.53	−0.19	0.03	0.03
	(1.64 to 1.72)	(1.45 to 1.59)	(−0.23 to −0.09)	(1.68 to 1.76)	(1.49 to 1.58)	(−0.23 to −0.14)	(−0.05 to 0.11)	(−0.04 to 0.11)
LDL, mmol/L	3.61	3.20	−0.41	3.61	3.17	−0.43	0.02	0.02
	(3.50 to 3.71)	(3.08 to 3.32)	(−0.54 to −0.28)	(3.51 to 3.70)	(3.06 to 3.29)	(−0.55 to −0.32)	(−0.13 to 0.18)	(−0.12 to 0.17)
WHR	0.89	0.87	−0.02	0.89	0.89	0.01	−0.02	−0.02
	(0.88 to 0.90)	(0.86 to 0.88)	(−0.03 to −0.01)	(0.88 to 0.89)	(0.89 to 0.90)	(0.00 to 0.02)	(−0.04 to −0.01)	(−0.03 to −0.01)

^a^: Effect size defined as the change for the intervention group minus the change for the control group; ^b^: Adjusted for work units, age, sex education, income, baseline value of variable.

In the intervention group, components of the 10-year risk of CVD (e.g., SBP, TC, BMI) changed significantly in the direction of decreased risk (all *p* < 0.05). In contrast, the control group presented a significant increase in SBP at follow-up (6.89 mmHg, 95%CI: 5.17 to 8.61, *p* < 0.001). Changes of TC and BMI were not statistically significant for participants in the control group. The differences of mean changes after adjustment for covariates in SBP, TC and BMI between study groups were −12.45 mmHg, −0.26 mmol/L and −0.86 kg/m^2^, respectively (all *p* < 0.05).

### 3.3. Secondary Study Outcomes

The mean value of DBP decreased at 12 months for the intervention group (–6.61 mmHg, 95%CI: –8.14 to –5.07, *p* < 0.001) and increased by 5.62 mmHg for the control group (95%CI: 4.39 to 6.84, *p* < 0.001). For FPG and WHR, there were significant reductions at 12 months for the intervention group (all *p* < 0.05). However, changes of FPG and WHR for participants in control group were not statistically significant. The HDL and LDL decreased at 12 months in both groups (all *p* < 0.05).

The differences of changes in secondary outcomes between intervention and control groups are shown in Table 3. Overall, the DBP, FPG and WHR changed significantly in the intervention group compared with the control group (DBP: −12.23 mmHg, 95%CI: −14.03 to −10.43, *p* < 0.001; FPG: –0.32 mmol/L, 95%CI: −0.51 to −0.13, *p* < 0.001; WHR: −0.02, 95%CI: −0.03 to −0.01, *p* < 0.001). The differences between the two groups in terms of TG, HDL and LDL were not statistically significant.

### 3.4. Subgroup Analysis and Sensitivity Analysis

**Figure 2:**
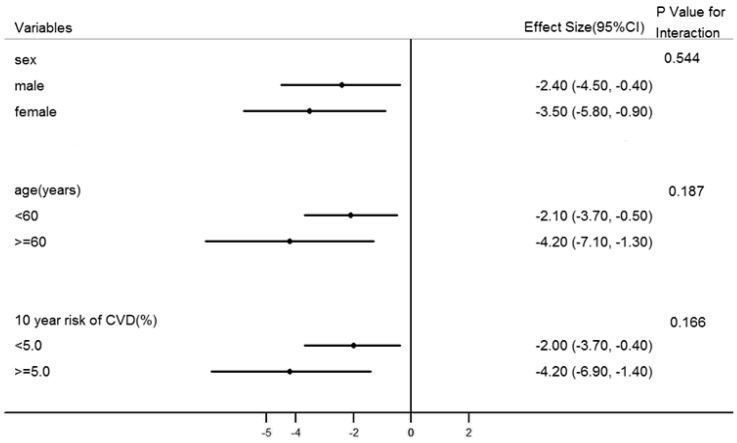
Effect size by subgroup analysis for 10-year risk of CVD.

Figure 2 displays the difference of mean changes in the 10-year risk of CVD between two groups stratified on selected baseline variables. In all subgroups, the effect size estimates were statistically significant. There were no significant interactions among the prespecified subgroups. A sensitivity analysis was conducted (Supplementary Table S3) on the completion population to assess the change in outcomes. Overall, results were similar, with significant difference of the changes in 10-year CVD risk between intervention and control groups.

## 4. Discussion

These results suggest that mobile phone-based lifestyle intervention, including CVD risk assessment and communication, and delivering tailored health education to subjects, could effectively slow down the increased estimated CVD risk with age when compared with the control group.

Results were similar across subgroups defined by baseline variables, and no differences were seen between subgroups. In addition, analyses of baseline and 1-year data showed statistically significant reductions in systolic and diastolic blood pressure, total cholesterol, BMI, fasting plasma glucose and waist hip ratio for the intervention group. These findings reinforce the increasing evidence suggesting that mobile phone-based intervention can play an important role in chronic disease prevention [24]. Just as mobile phones overcome barriers to communication, this technology is a promising tool to address several health care systems constraints in developing countries, such as limited financial resources, high population growth, a limited health care workforce, and difficulties in extending healthcare to hard-to-reach populations [10]. The mobile phone voice communication and text messaging were included as the intervention strategies in our study, as these are two main mobile phone functions currently used in China and other low- and middle- income countries. Previous studies indicated that interventions using short message service via mobile phone could improve several clinical outcomes (e.g., SBP, DBP, LDL, cholesterol) [25,26], processes of care (e.g., medication adherence) [27], and behavioral change (e.g., smoking cessation, weight loss) [28,29,30,31,32]. Peiris *et al.* proposed that the mobile phone would be widely used in primary health care settings for various purposes including data collection, health surveillance, health education, supervision, and monitoring [24]. In light of increasing disease burden of cardiovascular disease, mobile phone-based lifestyle intervention warrants further study to explore its effectiveness and cost-effectiveness for combating CVD and other chronic diseases in under-resourced settings.

Despite the public health relevance of estimated CVD risk, few studies of lifestyle interventions have used change in CVD risk as an outcome variable. Since cardiovascular disease is a result of the integration of multiple risk factors, interventions aimed to reduce the integrated risk are more appropriate in the cardiovascular disease prevention. Informing and communication with participants of their CVD risk could enhance their risk awareness and understanding of cardiovascular knowledge, so as to motivate them to take a healthier lifestyle and maintain with the medication [33,34]. In a previous study of 385 participants in North Carolina with 10-year coronary heart disease risk ≥ 10%, a lifestyle intervention reduced the 10-year coronary heart disease risk by approximately 2.0% [35]. Another randomized controlled trial of 315 participants with 10-year coronary heart disease risk ≥ 10% also reported a decrease in the risk score at 1-year follow-up for the lifestyle intervention (health report card with counselling on smoking, exercise, nutrition and stress) [9]. Although our study found no reduction in predicted 10-year CVD risk in both groups, it has shown that the mobile phone-based intervention could effectively slow down the increased predicted CVD risk with age when compared with the usual check group. This may be explained by the reason that the enrollment in our study included participants at all levels of 10-year CVD risk, other than the aforementioned studies, which recruited only those at moderate and/or high CVD risk level [9,17,35]. Subgroup analysis showed that the intervention had a greater impact on those with 10-year CVD risk ≥ 5% than those who weren’t. However, the *p* value for interaction was not significant. This finding might be caused by a lack of power for subgroup analyses.

Statistically significant improvements were observed between the intervention and usual check group for BP, TC, FPG, BMI and WHR. These findings could be an indication of the effectiveness of risk communication, follow-up phone calls and messages sending after medical examination. Moreover, risk communication, follow-up phone calls and messages sending after medical examination can be accomplished at a low cost, meaning that mobile phone-based intervention may be a worthwhile approach to adopt in the routine health examination.

This study has several limitations. Firstly, the generalizability of the findings may be limited. This study was undertaken in a medical examination center of a hospital in Guangzhou, and only involved the current or retired workers in work units. Therefore, our conclusions may be interpreted cautiously if extended to other populations. Secondly, the group-based randomization was applied to minimize the intervention contamination. However, the group randomization may have increased the possibility of unmeasured confounding variables because it was more likely to have resulted in differences between the two groups at baseline. Although our analyses included an adjustment for baseline differences in some potential confounders, it was impossible to adjust for all potential confounders. Thirdly, the participants in the control group only received a medical report with the results of physical examination and the normal values of the indicators, which were much less than would normally be expected following a check-up. This may partly explained the favorable effects of the intervention group. However, if the participants had any questions about their physical examination results, they would consult the doctors at the health management center. The doctors would provide a lifestyle suggestion or treatment recommendations to the participants. So the comparison between the intervention group and the controls was still meaningful. Fourthly, this study was not blinded but most of the outcomes were measured with an automated machine, such as the blood pressure, total cholesterol, triglyceride, fasting plasma glucose, high and low density lipoprotein cholesterol. This may allow the measurements to be taken without the need for intervention from the researcher once the machine turned on. Finally, 27.5% of participants failed to attend the follow-up. Participants who were lost to follow-up were more likely to be younger, male, current smokers and have a higher level of TC than those who were included in the follow-up. However, the sensitivity analyses suggest that these missing values are likely to have little impact on the primary outcome.

## 5. Conclusions

This study showed that mobile phone-based lifestyle intervention incorporated in the annual medical examination may be a potential solution for reducing cardiovascular disease risk among middle-aged and older adults in resources-limited settings, such as China. Further larger powered studies are needed to evaluate the effectiveness and cost-effectiveness of mobile phone-based interventions on cardiovascular risk prevention.

## Supplementary Files

Supplementary File 1
