# Supplementary material for: Mobile Phone-Based Lifestyle Intervention for Reducing Overall Cardiovascular Disease Risk in Guangzhou, China: A Pilot Study
Source: Int J Environ Res Public Health. 2015 Dec 17;12(12):15993–6004. doi: 10.3390/ijerph121215037 (PMC4690973; doi:10.3390/ijerph121215037)
Supplement: Supplementary File 1 [file ijerph-12-15037-s001.pdf]

Supplementary Information

Mobile Phone-Based Lifestyle Intervention for Reducing Overall Cardiovascular Disease Risk in Guangzhou, China: A Pilot Study

Table S1. Assessment of 10-year risk of ischemic cardiovascular disease (ICVD).

**Male**			
Step 1: Scoring The Cardiovascular Risk Factors			
Age	Score	SBP (mmHg)	Score
35~39	0	<120	−2
40~44	1	120~129	0
45~49	2	130~139	1
50~54	□	140~159	2
55~59	4	160~179	5
≥60, plus 1 score for each additional 5 years		≥180	8
BMI (kg/m^2)	Score	TC (mmol/L)	Score
<24	0	<5.17	0
≥24	2	≥5.17	1
Smoking	Score	Diabetes	Score
N	0	N	0
Y	2	Y	1
Step 2: Calculate the total score			
Step 3: Find the Corresponding Absolute Risk			
Total Score	Predicted 10-year risk (%)	Total Score	Predicted 10-year risk (%)
−2	0.3	8	7.0
−1	0.4	9	9.6
0	0.5	10	12.2
1	0.7	11	16.7
2	1.0	12	21.5
3	1.4	13	27.1
4	1.9	14	36.0
5	2.6	15	43.0
6	3.6	≥16	≥54.9
7	5.0		

Table S1. *Cont.*

**Male**			
Step 4: Compare with the Reference Standard			
Reference of predicted 10-year risk of ICVD			
Age	Average risk	Optimal risk	
35~39	0.9	0.3	
40~44	1.2	0.4	
45~49	1.6	0.5	
50~54	2.3	0.7	
55~59	3.1	1.0	
**Female**			
Step 1: Scoring The Cardiovascular Risk Factors			
Age	Score	SBP (mmHg)	Score
35~39	0	<120	−2
40~44	1	120~129	0
45~49	2	130~139	1
50~54	3	140~159	2
55~59	4	160~179	3
≥60, plus 1 score for each additional 5 years		≥180	4
BMI (kg/m^2)	Score	TC (mmol/L)	Score
<24	0	<5.17	0
≥24	2	≥5.17	1
Smoking	Score	Diabetes	Score
N	0	N	0
Y	1	Y	2
Step 2: Calculate the Total Score			
Step 3: Find the Corresponding Absolute Risk			
Total Score	Predicted 10-year risk (%)	Total Score	Predicted 10-year risk (%)
−2	0.1	7	3.3
−1	0.1	8	5.0
0	0.2	9	7.8
1	0.3	10	12.1
2	0.4	11	18.3
3	0.6	12	27.6
4	1.0	13	40.2
5	1.4	≥14	≥49.2
6	2.2		
Step 4: Compare with the reference standard			
Reference of predicted 10-year risk of ICVD			
Age	Average risk	Optimal risk	
35~39	0.2	0.1	
40~44	0.4	0.1	
45~49	0.6	0.2	
50~54	0.9	0.3	
55~59	1.3	0.5	

Note: The average risk refers to the average risk of the same age. The optimal risk denotes those who are non-smoker, non-diabetes of the same age and sex, with systolic blood pressure lower than 120 mmHg, total cholesterol lower than 5.17 mmol/L and body mass index lower than 24 kg/m^2.

Table S2. The comparison of main characteristics between participants lost to follow-up and retained (*n* (%)/Mean ± SD).

Characteristic	Retain	Lost	*p*
age	61.22 ± 8.22	58.84 ± 9.16	0.004
female	191 (44.7)	55 (34.0)	0.018
married	410 (96.0)	159 (98.2)	0.203
education			0.099
middle school or lower	90 (21.1)	46 (28.0)	
senior high school	111 (26.0)	32 (19.8)	
college or above	226 (52.9)	84 (51.9)	
personal monthly income			0.442
<¥3000	124 (29.0)	52 (32.1)	
¥3000~	112 (26.2)	47 (29.1)	
¥5000~	191 (44.7)	63 (38.9)	
current smoker	80 (18.7)	50 (30.9)	0.002
alcohol use	107 (25.1)	47 (29.0)	0.330
BMI, kg/m^2	23.98 ± 3.02	24.23 ± 3.36	0.375
WHR	0.89 ± 0.05	0.89 ± 0.06	0.753
SBP, mmHg	128.60 ± 13.47	127.00 ± 13.19	0.187
DBP, mmHg	78.59 ± 11.38	77.37 ± 9.90	0.202
FPG, mmol/L	5.56 ± 1.24	5.54 ± 1.48	0.908
TC, mmol/L	5.71 ± 1.01	5.45 ± 1.05	0.007
triglyceride, mmol/L	1.81 ± 1.21	1.79 ± 1.16	0.883
LDL, mmol/L	3.63 ± 0.89	3.54 ± 0.87	0.254
HDL, mmol/L	1.72 ± 0.36	1.66 ± 0.38	0.076
Hypertensive	112 (26.2)	33 (20.4)	0.141
Diabetic	30 (7.0)	16 (9.9)	0.250

Table S3. Sensitivity analyses based on the completion population.

Outcome	Intervention Group			Control Group			Crude Effect Size [a]	Adjusted Effect Size [b]
	Baseline	Year1	Change	Baseline	Year1	Change		
10-year risk of CVD	6.07 (4.88 to 7.26)	4.69 (3.58 to 5.79)	−1.35 (−1.97 to −0.74)	7.42 (6.50 to 8.34)	9.03 (7.94 to 10.13)	1.82 (1.12 to 2.51)	−3.13 (−4.15 to −2.12)	−3.15 (−4.12 to −2.18)
components of risk score								
SBP, mmHg	128.55 (126.26 to 130.85)	122.83 (120.54 to 125.11)	−5.55 (−6.93 to −4.42)	128.65 (127.14 to 130.15)	134.77 (132.98 to 136.40)	6.89 (4.91 to 7.23)	−11.75 (−13.53 to −9.97)	−11.76 (−13.48 to −10.04)
TC, mmol/L	5.65 (5.50 to 5.80)	5.29 (5.14 to 5.43)	−0.36 (−0.45 to −0.27)	5.74 (5.61 to 5.86)	5.53 (5.39 to 5.66)	−0.21 (−0.31 to −0.10)	−0.16 (−0.30 to 0.00)	−0.15 (−0.30 to −0.01)
BMI, kg/m^2	23.77 (23.28 to 24.25)	23.25 (22.78 to 23.72)	−0.57 (−0.67 to −0.46)	24.24 (23.76 to 24.46)	24.52 (24.16 to 24.89)	0.43 (0.36 to 0.50)	−0.99 (−1.12 to −0.87)	−0.99 (−1.12 to −0.87)
other outcomes								
DBP, mmHg	78.41 (76.78 to 80.05)	71.94 (70.34 to 73.53)	−6.64 (−7.58 to −5.70)	78.70 (77.27 to 80.13)	83.63 (82.40 to 84.85)	4.84 (4.00 to 5.67)	−11.49 (−12.79 to −10.12)	−11.49 (−12.69 to −10.29)
FPG, mmol/L	5.57 (5.36 to 5.78)	5.28 (5.10 to 5.45)	−0.30 (−0.39 to −0.21)	5.54 (5.40 to 5.68)	5.55 (5.42 to 5.69)	0.01 (−0.07 to 0.08)	−0.31 (−0.49 to−0.19)	−0.31 (−0.42 to −0.20)
TG, mmol/L	1.80 (1.58 to 2.03)	1.73 (1.51 to 1.94)	−0.09 (−0.28 to 0.11)	1.80 (1.68 to 1.92)	1.65 (1.54 to 1.77)	−0.15 (−0.25 to −0.04)	0.06 (−0.13 to 0.24)	0.06 (−0.12 to 0.25)
HDL, mmol/L	1.70 (1.65 to 1.76)	1.53 (1.47 to 1.58)	−0.18 (−0.22 to −0.13)	1.74 (1.69 to 1.78)	1.53 (1.49 to 1.58)	−0.20 (−0.23 to −0.17)	0.03 (−0.02 to 0.08)	0.03 (−0.02 to 0.07)
LDL, mmol/L	3.57 (3.45 to 3.69)	3.20 (3.08 to 3.32)	−0.36 (−0.46 to −0.26)	3.67 (3.56 to 3.79)	3.17 (3.06 to 3.29)	−0.49 (−0.58 to −0.41)	0.13 (0.00 to 0.26)	0.13 (0.01 to 0.25)
WHR	0.89 (0.88 to 0.89)	0.87 (0.86 to 0.88)	−0.01 (−0.02to 0.00)	0.89 (0.88 to 0.89)	0.89 (0.89 to 0.90)	0.01 (0.00 to 0.01)	−0.02 (−0.03 to −0.01)	−0.02 (−0.03 to −0.01)

[a]: Effect size defined as the change for the intervention group minus the change for the control group; [b]: Adjusted for work units, age, sex education, income, baseline value of variable

© 2015 by the authors; licensee MDPI, Basel, Switzerland. This article is an open access article distributed under the terms and conditions of the Creative Commons Attribution license (http://creativecommons.org/licenses/by/4.0/).
